# Proton‐Mediated and Ir‐Catalyzed Iron/Iron‐Oxide Redox Kinetics for Enhanced Rechargeability and Durability of Solid Oxide Iron–Air Battery

**DOI:** 10.1002/advs.202203768

**Published:** 2022-08-28

**Authors:** Qiming Tang, Chaitali Morey, Yongliang Zhang, Nansheng Xu, Shichen Sun, Kevin Huang

**Affiliations:** ^1^ Department of Mechanical Engineering University of South Carolina Columbia SC 29201 USA

**Keywords:** energy storage materials, long‐duration energy storage, rechargeability, reduction kinetics, reversibility

## Abstract

Long duration energy storage (LDES) is an economically attractive approach to accelerating clean renewable energy deployment. The newly emerged solid oxide iron–air battery (SOIAB) is intrinsically suited for LDES applications due to its excellent low‐rate performance (high‐capacity with high efficiency) and use of low‐cost and sustainable materials. However, rechargeability and durability of SOIAB are critically limited by the slow kinetics in iron/iron‐oxide redox couples. Here the use of combined proton‐conducting BaZr_0.4_Ce_0.4_Y_0.1_Yb_0.1_O_3_ (BZC4YYb) and reduction‐promoting catalyst Ir to address the kinetic issues, is reported. It is shown that, benefiting from the facilitated H^+^ diffusion and boosted FeO*_x_*‐reduction kinetics, the battery operated under 550 °C, 50% Fe‐utilization and 0.2 C, exhibits a discharge specific energy density of 601.9 Wh kg^–1^‐Fe with a round‐trip efficiency (RTE) of 82.9% for 250 h of a cycle duration of 2.5 h. Under 500 °C, 50% Fe‐utilization and 0.2 C, the same battery exhibits 520 Wh kg^–1^‐Fe discharge energy density with an RTE of 61.8% for 500 h. This level of energy storage performance promises that SOIAB is a strong candidate for LDES applications.

## Graphical abstract

Introducing proton conductor and active catalyst into nano‐Fe_3_O_4_ particles significantly boosts Fe_3_O_4_ reduction kinetics at intermediate temperature (500–550 °C), thus drastically improving the rechargeability of the solid oxide iron‐air battery (SOIAB). With this new energy storage unit (ESU) material, the SOIAB exhibits a remarkable 500‐h cycle life with a discharge energy density of 520 Wh kg^–1^‐Fe at 0.2 C and a round‐trip efficiency of 61.8%.

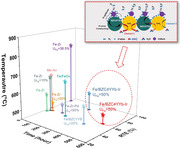

## 1 Introduction

The key to a successful deployment of clean renewable energy into the existing utility market is the development of reliable, low‐cost, and long‐duration energy storage (beyond daily cycles) technologies to compensate the intermittency of renewable energy production.^[^ ^1^, ^2^, ^3^ ^]^ The long duration energy storage (LDES) technology is particularly attractive due to its economic advantages.^[^ ^4^, ^5^ ^]^ Unfortunately, the most dominant large‐scale energy storage, for example, pumped hydro storage, is typically used for daily cycles. Conventional electrochemical batteries such as redox flow batteries, lithium‐ion batteries (LIBs), sodium–sulfur batteries, all have a cycle duration less than 10 h, not to mention the energy density and efficiency issues with redox flow batteries, and safety issues with LIBs and sodium–sulfur batteries.^[^ ^6^, ^7^, ^8^, ^9^ ^]^ Therefore, discovery and development of new battery chemistries with LDES capabilities are highly desirable for renewable industry.

**Scheme 1:**
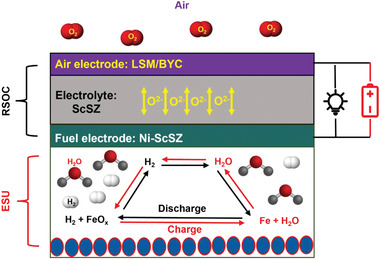
Components and working principle of solid oxide iron–air battery.

Solid oxide iron–air battery (SOIAB) is a newly emerged all solid‐state electrochemical cell, which has the potential to store large quantity of electricity within energy‐dense solid Fe at low cost and high efficiency.^[^ ^10^, ^11^, ^12^, ^13^, ^14^, ^15^, ^16^, ^17^ ^]^ This new battery consists of a reversible solid oxide cell (RSOC) and energy storage unit (ESU) containing an Fe/FeO*_x_* redox couple and H_2_/H_2_O oxygen shuttle; see **Scheme** **1**. Operated on the oxide–ion chemistry, SOIAB discharges electricity by oxidizing metallic Fe to FeO*_x_* and recharges itself by reducing FeO*_x_* to Fe. During the operation, RSOC acts as an electrical discharger and charger via solid oxide fuel cell and solid oxide electrolysis cell modes, respectively, while ESU serves as oxygen store (energy storage) with an H_2_–H_2_O mixture between RSOC's fuel electrode and ESU as the oxygen shuttle.^[^ ^10^ ^]^ During discharging; see black arrow in Scheme 1, the RSOC operates as a fuel cell model where the oxygen in the air electrode is reduced by electrons to O^2−^; the latter is then transported to the fuel electrode through the electrolyte and reacts with H_2_ in the ESU chamber to yield H_2_O. The produced H_2_O diffuses to ESU and oxidizes Fe, producing more H_2_ to sustain the electrochemical oxidation cycle. When the metal is fully or partly (in controlled portion) oxidized, the discharge process is stopped. During charging; see red arrow in Scheme 1, H_2_O in the ESU chamber is split into H_2_ and O^2−^ with electricity; the produced H_2_ diffuses back to ESU and reduces FeO*_x_* into Fe, producing more H_2_O to sustain electrochemical reduction cycle. When all FeO*_x_* (or a controlled portion) is depleted, the charging process is finished. Compared to the traditional metal–air batteries,^[^ ^18^ ^]^ one distinguished advantage of SOIAB is the decoupled energy and power. The energy capacity of SOIAB is exclusively determined by the mass of Fe in the ESU with the Fe‐utilization (*U*_Fe_) as the state of charge (SOC) indicator, while the power is solely dependent on the electrode surface area of RSOC. By scaling up Fe‐mass and RSOC size, an SOIAB system can store electricity in low‐cost Fe for days, weeks, months and even the entire renewable‐rich season.

Aimed at ensuring durability, a major development of SOIAB in recent years is to reduce the operating temperature to below 600 °C. Significantly improved durability has been demonstrated at reduced temperatures, but the power density has also been limited due to the lowered temperature.^[^ ^10^, ^19^ ^]^ For LDES operation; however, this is not necessarily a critical issue as LDES allows for low‐rate charging, that is, long cycle duration. What is important for the LDES systems is instead the capacity and efficiency. For SOIAB to be a LDES‐compatible system, the reversibility of ESU, or specifically the FeO*_x_* reduction kinetics that determines the rechargeability of the battery, needs to be further enhanced. We have previously discussed and demonstrated this critical issue theoretically and experimentally.^[^ ^20^, ^21^ ^]^

Our early effort to improve the FeO*_x_*‐reduction kinetics includes the use of notable metals such as Pd as catalyst^[^ ^10^ ^]^ and proton conducting oxide as the support for Fe particles.^[^ ^17^ ^]^ While these efforts have resulted in improved battery performance, there is still a performance gap for practical applications. Here, we report on our recent efforts to further raise SOIAB performance toward practical applications. A focus of the work is to combine a new catalyst with proton conducting oxide support in ESU to substantially boost the FeO*_x_*‐reduction kinetics, thus increasing round‐trip efficiency (RTE) or reversibility (rechargeability). Compared to the baseline ESU (Fe_2_O_3_/ZrO_2_) material, we show that the newly developed ESU material exhibits superior catalytic activity toward FeO*_x_*‐reduction kinetics and enables the SOIAB to operate at 500–550 °C with excellent stability and high RTE.

## 2 Results and Discussion

### 2.1 Phase Composition and Morphologies of ESU Materials

**Figure 1:**
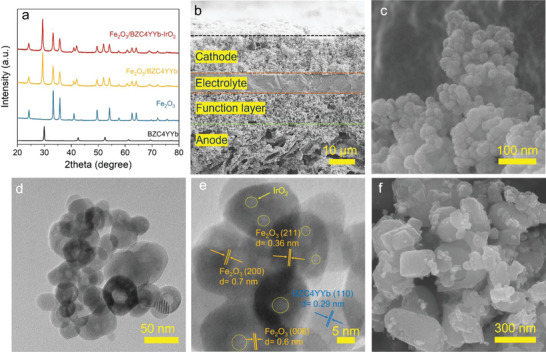
a) XRD patterns of as‐prepared samples; b) cross‐sectional views of RSOC; c) SEM images of as‐prepared Fe/BZC4YYb/Ir; d) TEM image of Fe/BZC4YYb/Ir; e) HRTEM image of Fe/BZC4YYb/Ir; f) SEM images of Fe/BZC4YYb/Ir after 250 hours testing with *U*_Fe_ = 50% and 0.2 C at 550 °C.

The phase purity of the as‐prepared BaZr_0.4_Ce_0.4_Y_0.1_Yb_0.1_O_3_ (BZC4YYb) was confirmed by XRD patterns shown in **Figure** **1**a, where all the diffraction peaks match with the literature data.^[^ ^22^ ^]^ After mixing with Fe_2_O_3_, there are no extra peaks found, suggesting that there is no reaction between BZC4YYb and Fe_2_O_3_, which is expected. There is no additional phase observed either after infiltrating IrO_2_. In addition, the XRD pattern of the tested sample shown in Figure S1, Supporting Information indicates that BZC4YYb still maintains its original phase, whereas iron oxide has been reduced into metallic iron.

The cross‐sectional view of the RSOC as SEM image after reduction in H_2_‐3% H_2_O is shown in Figure 1b. A 10‐µm thick, dense ScSZ electrolyte and 15‐µm thick, porous fuel electrode‐functional layer are clearly seen on the fuel electrode‐support. The LSM/BYC air electrode screen‐printed on the ScSZ surface is 18 µm thick. Uniform pores in a diameter of ≈5 µm are also seen to distribute across the fuel electrode, which ensures facile H_2_ and H_2_O diffusion. Figure S2a,c, Supporting Information, further shows the morphologies of as‐prepared Fe/ZrO_2_ baseline and Fe/BZC4YYb ESU materials, both of which exhibit an average grain size of ≈30 nm. Note that we use Fe instead of Fe_2_O_3_ hereafter in the presentation of battery performance to reflect the fact that Fe is the true active ESU material.

Figure 1c,d shows a uniform morphology of Fe/BZC4YYb. After impregnating IrO_2_ nanoparticles (NPs), Figure 1e shows HRTEM images of IrO_2_ (NPs) in a size of 5 nm. With elemental mapping (Figure S3, Supporting Information) and EDS results (Figure S4, Supporting Information), it is obvious that IrO_2_ NPs are uniformly distributed across Fe/BZC4YYb particles with an estimated Ir content of 1.9 wt%. After 250 h testing with *U*_Fe_ = 50% and 0.2 C at 550 °C, the Fe/BZC4YYb/Ir only exhibits a slight coarsening; see Figure 1f; Figure S5, Supporting Information. In contrast, visible agglomerations for baseline and Fe/BZC4YYb are observed in Figure S2b,d, Supporting Information, after cycling.

### 2.2 Evaluation of Electrochemical Performance

**Figure 2:**
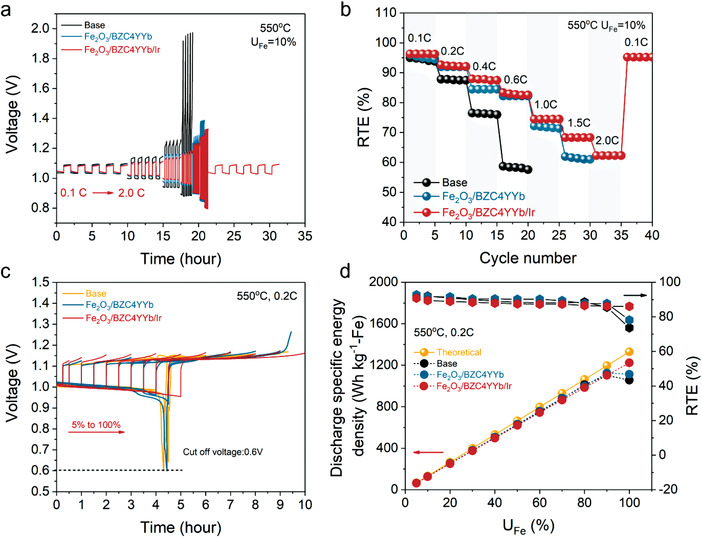
Electrochemical performance of SOIAB with different ESU materials: a) voltage profiles versus time under different current density with a fixed *U*_Fe_ = 10%; b) RTE versus C‐rate; c) voltage profiles versus time at a fixed *j* = 10 mA cm^–2^ (0.2 C) and different *U*_Fe_ (5–100%); d) discharge specific energy density and corresponding RTE versus *U*_Fe_.

The details of battery fabrication process are provided in the Experimental Section. Note that the starting active material is Fe_2_O_3_. Upon H_2_‐reduction, it transforms to metallic Fe. The discharge cycle is first performed on metallic Fe with oxygen being transported into ESU chamber, while establishing Fe/Fe_3_O_4_–H_2_O/H_2_ equilibrium. The overall performance of SOIAB with different ESU material is summarized in **Figure** **2** **;** Figure S6, Supporting Information. Specifically, Figure 2a,b compares the voltage profiles versus time and the corresponding RTE at a fixed *U*_Fe_ = 10% but with different C‐rates for the three ESU materials. The results show that Fe/BZC4YYb can achieve 1.5 C (75 mA cm^–2^) at RTE ≈ 60.6% versus 0.6 C (30 mA cm^–2^) at RTE ≈ 58% for the baseline Fe/ZrO_2_, demonstrating improved rate performance by incorporating an H^+^ conductor in the ESU. After adding Ir (decomposed from IrO_2_) into Fe/BZC4YYb, the rate performance is substantially enhanced; at 2.0 C (100 mA cm^–2^), the battery can achieve a high RTE = 62.3%. The performance is also reversible as C‐rate returns to 0.1 C; a high RTE = 95.2% is still achievable.

The benefit of combining Ir with BZC4YYb to improve the kinetics of ESU is also illustrated in Figure 2c, where battery's voltage profile is measured versus *U*_Fe_. For the baseline and Fe/BZC4YYb, the discharge voltage experiences a sharp drop at *U*_Fe_ = 80% and *U*_Fe_ = 90%, respectively, while the charging voltage exhibits less changes. These rapid drops of discharge voltage are attributed to the diffusion limitation caused by the formation of thick and dense Fe_3_O_4_ layer at high *U*_Fe_. In contrast, Fe/BZC4YYb/Ir yields a stable discharge as well as charge voltage even at *U*_Fe_ = 100%, suggesting that it significantly enhances the catalytic activity toward Fe/FeO*_x_* redox reaction.

The specific energy density and RTE are further calculated from Figure 2c and shown in Figure 2d versus *U*_Fe_. As expected, discharge specific energy density (DSED) increases with *U*_Fe_, for example, it reaches 251.5, 621.3, and 1223.7 Wh kg^–1^‐Fe at *U*_Fe_ = 20%, 50%, and 100%, respectively, for Fe/BZC4YYb/Ir ESU; the corresponding RTEs are 91.5%, 90.2%, and 78.2%. It is worth noting that *U*_Fe_ has a smaller effect on RTE than that of C‐rate. This unique observation again suggests that SOIAB is better suited for low C‐rate and high *U*_Fe_ operation, such as LDES.

### 2.3 Long‐Term Performance Evaluation

The long‐term cycling performance of the battery with three different ESU materials was first evaluated at 550 °C and at low 0.2 C (10 mA cm^–2^) but different *U*_Fe_; the results are shown in Figure S7a,b, Supporting Information. The stability and RTE with Fe/BZC4YYb (with or without Ir catalyst) are slightly better than the baseline ESU, thus reflecting some benefits of H^+^ conducting materials. The partial agglomeration of the baseline ESU shown in Figure S2b, Supporting Information, could be another reason for the difference. Nevertheless, it is clear that the addition of BZC4YYb and Ir does not significantly increase the battery performance at such a low *U*_Fe_. In other words, the redox reaction within Fe/FeOx couple is kinetically fast enough to match RSOC's current density under low *U*_Fe_, which is also reflected in its high RTE = 87.4%. However, the battery's energy density is low under low *U*_Fe_, for example, it only delivers DSED ≈ 60.2 Wh kg^–2^‐Fe. Therefore, demonstration of long‐term cycle stability under high *U*_Fe_, thus high energy capacity, is practically more meaningful. We, therefore, continued testing the battery under higher *U*_Fe_.

**Figure 3:**
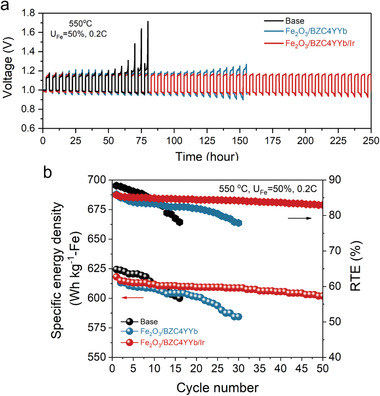
Cycling performance of a 550 °C battery with different ESU materials at *U*_Fe_ = 50% and 0.2 C (10 mA cm^–2^): a) voltage profiles; b) the corresponding specific energy density (SED) and RTE.

Under the condition of 550 °C, *U*_Fe_ = 50% and 0.2 C with 2.5 h cycle duration; **Figure** **3**a shows the voltage profiles versus cycle time of the battery loaded with baseline, Fe/BZC4YYb and Fe/BZC4YYb/Ir ESU materials. It is evident that Fe/BZC4YYb/Ir exhibits the best stability, over 250 h (or 50 cycles) without significant degradation. The corresponding DSED and RTE shown in Figure 3b are DSED = 601.9 Wh kg^–1^ and RTE = 82.9%, respectively. In contrast, RTE of the battery with the baseline ESU faded rapidly to 75% only after 15 cycles, while the battery with Fe/BZC4YYb ESU shows RTE ≈ 80% after 30 cycles. This comparison provides additional evidence that both BZC4YYb and Ir contribute to the cycle stability at high *U*_Fe_.

**Figure 4:**
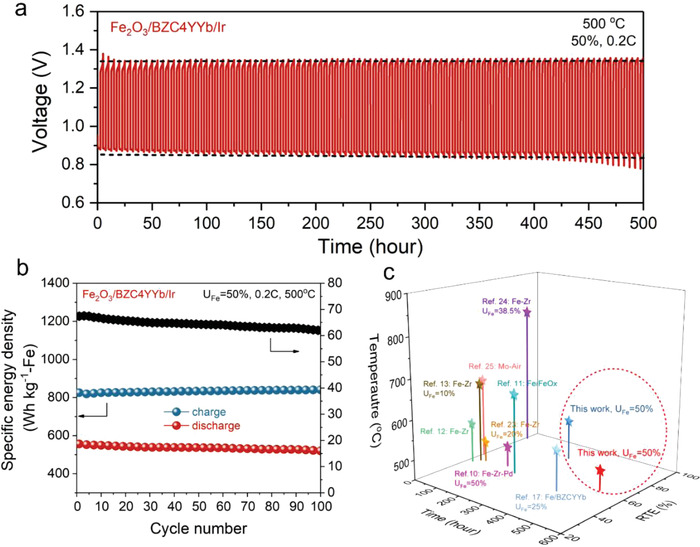
Cycling performance of a 500 °C‐battery with Fe/BZC4YYb/Ir ESU materials at *U*_Fe_ = 50% and 0.2 C (10 mA cm^–2^): a) voltage profiles; b) the corresponding SED and RTE; and c) comparison with previous reports.^[^ ^10^, ^11^, ^12^, ^13^, ^17^, ^23^, ^24^, ^25^ ^]^

To further demonstrate the battery performance at lower temperature, the same battery with the best Fe/BZC4YYb/Ir ESU material was further tested under the condition of 500 °C, *U*_Fe_ = 50% and 0.2 C. **Figure** **4**a shows the voltage profiles versus cycle time. The battery can cycle for additional 500 h without significant degradation. The corresponding DSED and RTE shown in Figure 4b are DSED = 520 Wh kg^–1^ and RTE = 61.9%, respectively. The lower operating temperature clearly benefits the overall long‐term cycle stability, but the efficiency of battery is negatively affected, which is understandable because the electrochemical performance of RSOC and redox kinetics are lower at 500 °C than 550 °C; Figure S8, Supporting Information provides the RSOC performance at 500 °C. Compared to previous reports (Figure 4c), this new ESU material shows the best durability and efficiency in the intermediate temperature range.^[^ ^10^, ^11^, ^12^, ^13^, ^17^, ^23^, ^24^, ^25^ ^]^

### 2.4 Understanding the Beneficial Role of BZC4YYb/Ir Toward FeO*_x_* Reduction Kinetics

To understand the role of H^+^ and Ir in catalyzing FeO*_x_* reduction, we performed temperature‐programmed reduction (TPR) studies on the as‐prepared baseline, Fe_2_O_3_/BZC4YYb and Fe_2_O_3_/BZC4YYb/Ir in 10%H_2_–Ar. The TPR profiles for different samples are shown in Figure S9, Supporting Information. For the baseline Fe_2_O_3_/ZrO_2_, there are three well‐separated reduction peaks; the low temperature peak (304.5 °C) reflects the first stage of the reduction of hematite to magnetite *α*‐Fe_2_O_3_ → Fe_3_O_4_ and the medium temperature peak (484.5 °C) is assigned to the reduction of Fe_3_O_4_ → FeO. The third peak, broad and much larger, at 605.3 °C, represents the reduction of wurtzite to metallic iron, FeO → Fe. For Fe_2_O_3_/BZC4YYb, the main reduction peak of Fe_2_O_3_ shifts from 605.3 °C to 564.3 °C, suggesting the enhanced reduction kinetics. After introducing IrO_2_, the reduction peaks continually move to lower temperature. It is worth noting that the first peak at 136.5 °C is due to the reduction of IrO_2_ to Ir. Only two peaks can be observed in the next reduction: the first one at 242.5 °C is related to Fe_2_O_3_–Fe_3_O_4_ and the second one at 525.4 °C is associated with the direct Fe_3_O_4_ reduction to Fe, suggesting an enhanced reduction kinetics without or with minimal amount of the intermediate FeO phase.

With increasing the temperature ramping rate, all the reduction peaks experienced a right shift, which is caused by the diffusion limitation at high ramping rates. Figure S10, Supporting Information shows the TPR profiles collected at different ramping rates. According to the Kissinger's method, the linear relationship between $\ln(\frac{\Phi }{T_{\max}^{2}})$ versus $\frac{1}{T_{\max}}$ is plotted in Figure S11, Supporting Information. From the slope of each line, the activation energy for Fe_2_O_3_/BZC4YYb/Ir is 32.8 kJ mol^–1^ for the direct Fe_3_O_4_ to Fe reduction, while it is 57.1 kJ mol^–1^ for Fe_3_O_4_ to FeO reduction and 74.6 kJ mol^–1^ for FeO to Fe reduction, respectively, with Fe_2_O_3_/BZC4YY, which are all lower than those of baseline.^[^ ^17^ ^]^

**Figure 5:**
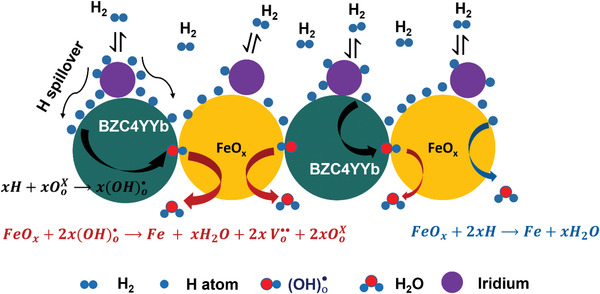
Schematic illustration of reduction process in H_2_ over proton conducting BZC4YYb support and Ir catalyst.

To elucidate mechanistically the promotional effect of BZC4YYb and Ir on FeO*_x_* reduction kinetics, **Figure** **5** schematically illustrates the H_2_ reduction process over Fe/BZC4YYb/Ir. The Ir NPs are known to be catalytic to H_2_ spillover.^[^ ^26^, ^27^, ^28^, ^29^ ^]^ Therefore, Ir‐enhancement on FeO*_x_* reduction kinetics is well‐expected. However, how H^+^ conducting support BZC4YYb promotes the reduction kinetics needs further explanation.

We note that there are two parallel reaction pathways for H diffusion in Fe/BZC4YYb. On the one hand, the conventional reduction reaction of FeO*_x_* to Fe and release water takes place at the surface of FeO*_x_* particle during the charging process, via

(1) $${\mathrm{FeO}}_{x}+2xH\to \mathrm{Fe}+xH_{2}O$$

On the other hand, a parallel reduction process also occurs at the interface of BZC4YYb and FeO*_x_*, which consists of the following two elementary steps:

1. Transport of the dissociated H through BZC4YYb bulk to the interface with FeO*_x_*:
(2) $$2xH+2x{\;V}_{o}^{••}+2xO_{o}^{X}\to 2x{\left(\mathrm{OH}\right)}_{o}^{•}$$
2. Chemical reduction of FeO*_x_* to Fe and generation of water:
(3) $${\mathrm{FeO}}_{x}+2x{\left(\mathrm{OH}\right)}_{o}^{•}\to \mathrm{Fe}\;+\;xH_{2}O\left(g\right)+2x{\;V}_{o}^{••}+2xO_{o}^{X}$$

Here, we use Kröger–Vink notation to describe the defects involved. ${\;V}_{o}^{••}$, $O_{o}^{X}$, and ${\left(\mathrm{OH}\right)}_{o}^{•}\;$represent oxygen vacancy, lattice oxygen, and proton on oxygen lattice, respectively.

A reverse process is expected for the oxidation process (discharge) in Fe/BZC4YYb:

(4) $$H_{2}O\left(g\right)+{\;V}_{o}^{••}+O_{o}^{X}\to 2{\left(\mathrm{OH}\right)}_{o}^{•}$$

(5) $$\mathrm{Fe}+2x{\left(\mathrm{OH}\right)}_{o}^{•}\to {\mathrm{FeO}}_{x}\;+\;xH_{2}+x{\;V}_{o}^{••}+xO_{o}^{X}$$

Thus, through the production and transport of ${\left(\mathrm{OH}\right)}_{o}^{•}$, additional proton pathways could be provided to facilitate the overall reduction kinetics.

## 3 Conclusion

In summary, a combination of proton conducting BCZ4YYb support and Ir‐catalyst in Fe‐based ESU material has resulted in a significantly enhanced FeO*_x_*‐reduction kinetics; thus, reversibility and durability of SOIAB. At 550 °C, the battery with the new ESU explicitly outperforms the battery with the baseline Fe/ZrO_2_ ESU, capable of operating at a C‐rate as high as 2C (100 mA cm^–2^) with a RTE of 62.3%. Under LDES‐related working conditions, for example*, U*_Fe_ = 50% and 0.2 C, the battery can achieve a discharge energy density of 601.9 Wh kg^–1^‐Fe for 50 cycles (250 h at 2.5 h cycle duration) with an RTE of 82.9%. At 500 °C, the same battery can still deliver a discharge specific energy density as high as 520 Wh kg^–1^‐Fe at a RTE of 61.8%, with a substantially enhanced durability, that is, 100 cycles (500 h at 2.5 h cycle duration). With the performance presented in this study, we strongly believe that SOIAB technology is technically suited for LDES applications in the future.

## 4 Experimental Section

### Materials Synthesis

*Fe_2_O_3_/ZrO_2_ Baseline ESU*: It was prepared by a co‐precipitation method. Briefly, stoichiometric Fe(NO)_3_•9H_2_O (≥99.999%, Sigma–Aldrich) and ZrO(NO)_2_•*x*H_2_O (≥99.999%, Alfa‐Aesar, *x* = 2) were first dissolved in deionized water separately. Then, the two solutions were mixed in a beaker with a cation concentration of 0.1 m. The mixture solution was then added dropwise to 1 L (NH_4_)_2_CO_3_ (Sigma–Aldrich) solution bath under constant stirring. To ensure a full precipitation of all cations in the solution, the molar ratio of (NH_4_)_2_CO_3_ and Me^+^ (*M* = Zr and Fe) was kept as *n* (NH_4_)_2_CO_3_ : *n* Me^+^ = 1.5 : 1. The resultant brownish precipitate was then left in the solution for 20 h with continuous stirring. Finally, the aged suspension was filtered and washed several times with DI water, dried overnight at 80 °C and calcined in air at 600 °C for 5 h to yield the final product.

*Fe_2_O_3_/BZC4YYb ESU*: The BaCe_0.4_Zr_0.4_Y_0.1_Yb_0.1_O_3‐*δ*_ (BZC4YYb) powders were prepared by a solid‐state reaction (SSR) method. Briefly, BaCO_3_ (Sigma–Aldrich, ACS Reagent, ≥99%), CeO_2_ (Acro's Organics, 99.9%), ZrO_2_ (Alfa Aesar, 99.7%), Y_2_O_3_ (Alfa Aesar, 99.9%), and Yb_2_O_3_ (Sigma–Aldrich, 99.9%) were intimately mixed according to their stoichiometries with ethanol in a milling jar for 24 h, followed by drying in an oven and calcining at 900 °C for 10 h in air. The powders were then ball milled by a micronizing mill (McCrone) for 30 min, followed by pressing into a disk and sintering at 1350 °C for 10 h. Extra powders were used to cover the pellets to prevent and compensate Ba‐loss during sintering. This milling–pelletizing–sintering process was repeated twice to achieve a pure perovskite phase. Finally, 3.25 g Fe_2_O_3_ nanoparticles (99.5%, US Research Nanomaterials, Inc.) and 1.1 g BZC4YYb powders were mixed/milled for 20 h with ethanal (100%, Sigma–Aldrich) as medium in a planetary ball mill (BM4X‐04, COL‐INT TECH) using ZrO_2_ balls and container at a milling speed of 300 rpm. The mixture was dried in 80 °C and ready for use.

*Fe_2_O_3_/BZC4YYb‐IrO_2_ ESU*: Fe_2_O_3_/BZC4YYb‐IrO_2_ was prepared by infiltration method. Briefly, 0.1 g Ir precursor, iridium III 2,4‐pentanedionate (C_15_H_21_IrO_6_, Sigma–Aldrich), dissolved in 2 mL acetone (99.5%, Sigma–Aldrich), was added into 1 g of ball‐milled Fe_2_O_3_/BZC4YYb powders in an agate mortar, followed by thorough mixing. Finally, the powder was calcined at 600°C for 2 h. The total IrO_2_ loading in the ESU was ≈4 wt% of the Fe_2_O_3_/BZC4YYb mass.

*La_0.8_Sr_0.2_MnO_3_ (LSM)/(Bi_0.75_Y_0.25_)_0.93_Ce_0.07_O_1.5_ (BYC) Air Electrode*: It was prepared by combustion method using nitrates as the metal precursors. Briefly, for LSM preparation, stoichiometric amounts of La(NO_3_)_3_·6H_2_O (Sigma–Aldrich), Sr(NO_3_)_2_ (Sigma–Aldrich), and Mn(NO_3_)_2_·4H_2_O (Sigma–Aldrich) were first dissolved into 0.2 m citric acid (CA, Sigma–Aldrich) solution with a molar ratio of metal ions:CA = 1:2. Then, 10 mL nitric acid (70%, Sigma–Aldrich) solution was added into the solution while stirring. The pH of the solution was then adjusted to ≈6 with ammonia (28–30%, Sigma–Aldrich). Finally, the transparent solution was gradually heated in an oven at 240 °C until auto‐combustion. The obtained powders were then broken up and calcinated at 900 °C for 5 h to form the phase. The BYC powders were prepared by a similar process with Bi(NO_3_)_3_·5H_2_O (Sigma–Aldrich), Y(NO_3_)_3_·6H_2_O (Sigma–Aldrich), and Ce(NO_3_)3·6H_2_O (Sigma–Aldrich) as the metal precursors, except that the molar ratio of the metal ions to CA was 1:1.5 and the calcination temperature was 700 °C.

### Battery Fabrication, Assembly, and Testing

For RSOC, the fuel electrode‐supported electrolyte half‐cell was fabricated by dry‐pressing and dip‐coating method as described before.^[^ ^30^ ^]^ Briefly, a mixture of NiO (Inframat Advanced Materials), (Sc_2_O_3_)_0.1_(CeO_2_)_0.01_(ZrO_2_)_0.89_ (ScSZ, Daiichi Kigenso Kagaku Co. Ltd., Japan), and graphite with a weight ratio of 60:40:30 was mixed with ethanol in a planetary mill for 2 h followed by drying at 80 °C overnight. The dried powders were then fully mixed with 5 wt% PVB in acetone with an agate mortar, followed by pressing into 𝛷1.0′′ pellets and partially sintering at 900 °C for 2 h to achieve enough strength. A NiO/ScSZ functional layer (≈15 µm, NiO:ScSZ = 60:40 in weight) and ScSZ electrolyte layer (≈10 µm) were subsequently dip‐coated on the fuel electrode‐support using a slurry. The multilayer structure was finally sintered at 1350 °C for 5 h to achieve dense ScSZ electrolyte. Then, an ink made of LSM/BYC and V‐006 binder (Columbia International) in a weight ratio of LSM:BYC:V‐006 = 40:60:150 was screen printed on top of ScSZ electrolyte supported on fuel electrode substrate. The whole assembly was then calcined at 800 °C for 2 h. The fuel electrode consisted of NiO and ScSZ porous structure infiltrated with 2 wt% GDC nanoparticles.

To assemble the battery, the prepared ESU materials in the amount of ≈0.1 g (with 0.01 mol Fe element) were first placed in the battery holder, followed by placing the RSOC on top of a specially designed Inconel holder and sealing with glass powder (melting point: 680 °C, Schott GM31107). The battery was then gradually ramped up to 680 °C to melt the glass and then cooled down 500–550 °C for testing. Before testing, the Fe_2_O_3_ in the ESU was first reduced into active metallic Fe with a 5%H_2_–N_2_ mixture for 10 h. The testing of electrochemical performance of the battery was carried out with a Solartron Multichannel system (model 1470e) coupled with a Solartron 1255 frequency response analyzer (FRA). The OCV, *V*–*j* curves and electrochemical impedance spectroscopy (EIS) of the RSOC were first measured for both open (flowing H_2_) and close systems. Then, the performance under different rates and *U*_Fe_ were systematically measured. The cutoff voltages for discharge and charge were set to 0.6 and 2.0 V, respectively. Detailed testing procedure can be found in our early works.^[^ ^10^ ^]^

### Materials Characterization

The phase compositions of the prepared ESU materials were examined by X‐ray diffraction (XRD, Rigaku D/MAX‐2100) at a scan rate of 5° min^–1^ from 10° to 80°. The morphologies of the ESU materials and battery cell components were examined by an ultra‐high resolution field emission scanning electron microscope (UHR‐FESEM) (HITACHI, Regulus 8230) equipped with energy dispersive X‐ray spectroscopy (EDS). A high‐resolution transmission electron microscope (HRTEM, HITACHI H‐9500) and scanning transmission electron microscope (STEM, HITACHI SU9000) were also used to examine the elemental distribution. Temperature‐programmed reduction (TPR) experiments were performed with a Micromeritics Chemisorption Analyzer (model 2720) in 10% H_2_/Ar to study the reduction kinetics of ESU materials. An ≈30 mg as‐prepared ESU material sample was placed in a quartz tube and data were collected at different ramping rates of 2.5 °C, 5 °C, 10 °C, and 15 °C min^−1^ as the temperature was increased from 100 °C to 900 °C.

### Statistical Analysis

Statistical analysis was carried out using OriginLab 2017 (OriginLab Corp., Northampton, USA) and Microsoft Excel (Microsoft Corp., USA). It included the following components.

*Pre‐Processing of Data*: The voltage and current were directly collected from potentiostat (Solartron 1287 Electrochemical Interface) with a precision of ±1 µV and ±1 µA, respectively. Energy capacity was calculated based on voltage and current data.

*Data Presentation*: All voltage and current data presented; therefore, have a standard deviation of ±1 µV and ±1 µA, respectively.

*Sample Size*: There are hundreds of data points collected during cycling test.

*Statistical Methods*: No specific statistical testing was performed in this study as the level of energy capacity is of paramount consideration given the high precision of the potentiostat employed.

### Electrochemical Performance Calculations

At 550 °C, the equilibrium between Fe and O_2_ in SOIAB was given by:^[^ ^31^, ^32^ ^]^

(6) $$3Fe\left(s\right)+4O_{2}\left(g\right)\Leftrightarrow Fe_{3}O_{4}\left(s\right)$$

The theoretical Nernst potential (*E*_N_) related to this reaction was determined by:

(7) $$E_{N}=E_{o}\;-\frac{RT}{nF}\ln\left(\frac{1}{{\left[P_{O_{2}}\right]}^{4}}\right)$$

where $E_{o}=\;-\frac{\Delta G^{0}}{nF}$, which refers to the EMF under the standard state; Δ*G* ^0^ is the standard Gibbs free energy change of Fe–Fe_3_O_4_ reaction at 550 °C; R is the universal gas constant, 8.314 J mol^–1^ K^–1^; *T* is the temperature in kelvins; *n* is the number of electrons transferred in the reaction, *n* = 8; F is the Faraday constant, 96 485 C mol^–1^; and $P_{O_{2}}$ is the partial pressure of oxygen, 0.21 atm in air.

Thus, the theoretical specific energy (SE*, based on the mass of iron element, Wh kg^–1^) can be calculated by:

(8) $${\mathrm{SE}}^{*}\;\;\left({\mathrm{Wh}\;\mathrm{kg}}^{-1}\right)=\frac{Q^{*}\times E_{N}}{m_{\mathrm{Fe}}\left(\mathrm{kg}\right)}\;=\frac{nF\times E_{N}}{3600\;\times M_{\mathrm{Fe}}\times 0.001}\;$$

where Q* = *n* × F = *I* × *t*(*s*), which refers to the total theoretical charge per mole of Fe; *M*_Fe_ is the molar mass of Fe in g mol^–1^.

The practical specific energy (SE, based on the mass of iron element) is determined by the following:

(9) $$\mathrm{SE}\;\;\left({\mathrm{Wh}\;\mathrm{Kg}}^{-1}\right)=\frac{\int_{t_{1}}^{t_{2}}\left(I\times V\right)dt}{m_{\mathrm{Fe}}\left(\mathrm{kg}\right)}\;$$

where, *t*_1_ and *t*_2_ refers to the duration of charging or discharging, h, respectively; *I* is the applied current, A; and *V* is the actual measured potential, volt.

## Conflict of Interest

The authors declare no conflict of interest.

## Supporting information

Supporting Information

## Data Availability

The data that support the findings of this study are available from the corresponding author upon reasonable request.
