# Supplementary material for: Proton‐Mediated and Ir‐Catalyzed Iron/Iron‐Oxide Redox Kinetics for Enhanced Rechargeability and Durability of Solid Oxide Iron–Air Battery
Source: Adv Sci (Weinh). 2022 Aug 28;9(30):2203768. doi: 10.1002/advs.202203768 (PMC9596828; doi:10.1002/advs.202203768)
Supplement: Supplementary file 1 — Supporting Information [file ADVS-9-2203768-s001.pdf]

Supporting Information

Proton-Mediated and Ir-Catalyzed Iron/Iron-Oxide Redox Kinetics for Enhanced Rechargeability and Durability of Solid Oxide Iron-Air Battery

*Qiming Tang, Chaitali Morey, Yongliang Zhang, Nansheng Xu, Shichen Sun and Kevin Huang**

Department of Mechanical Engineering, University of South Carolina, Columbia, SC 29201, USA

*Corresponding author: e-mail: *huang46@cec.sc.edu*

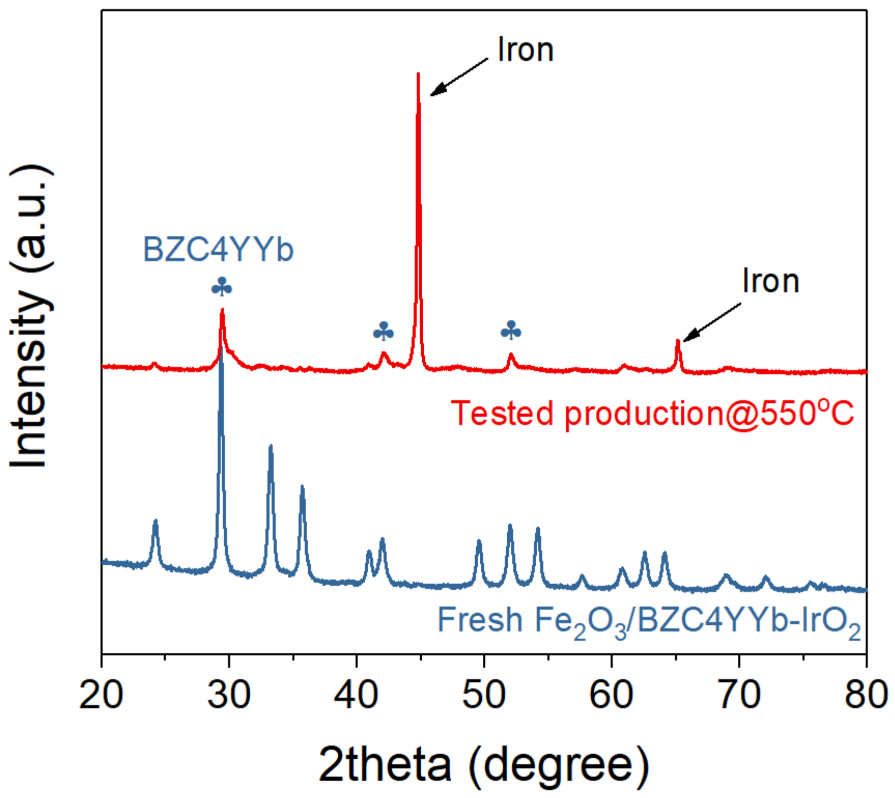

Figure S1 XRD pattern of sample after 250 hours testing at U_{Fe}=50% and 0.2C at 550 ^{o}C.

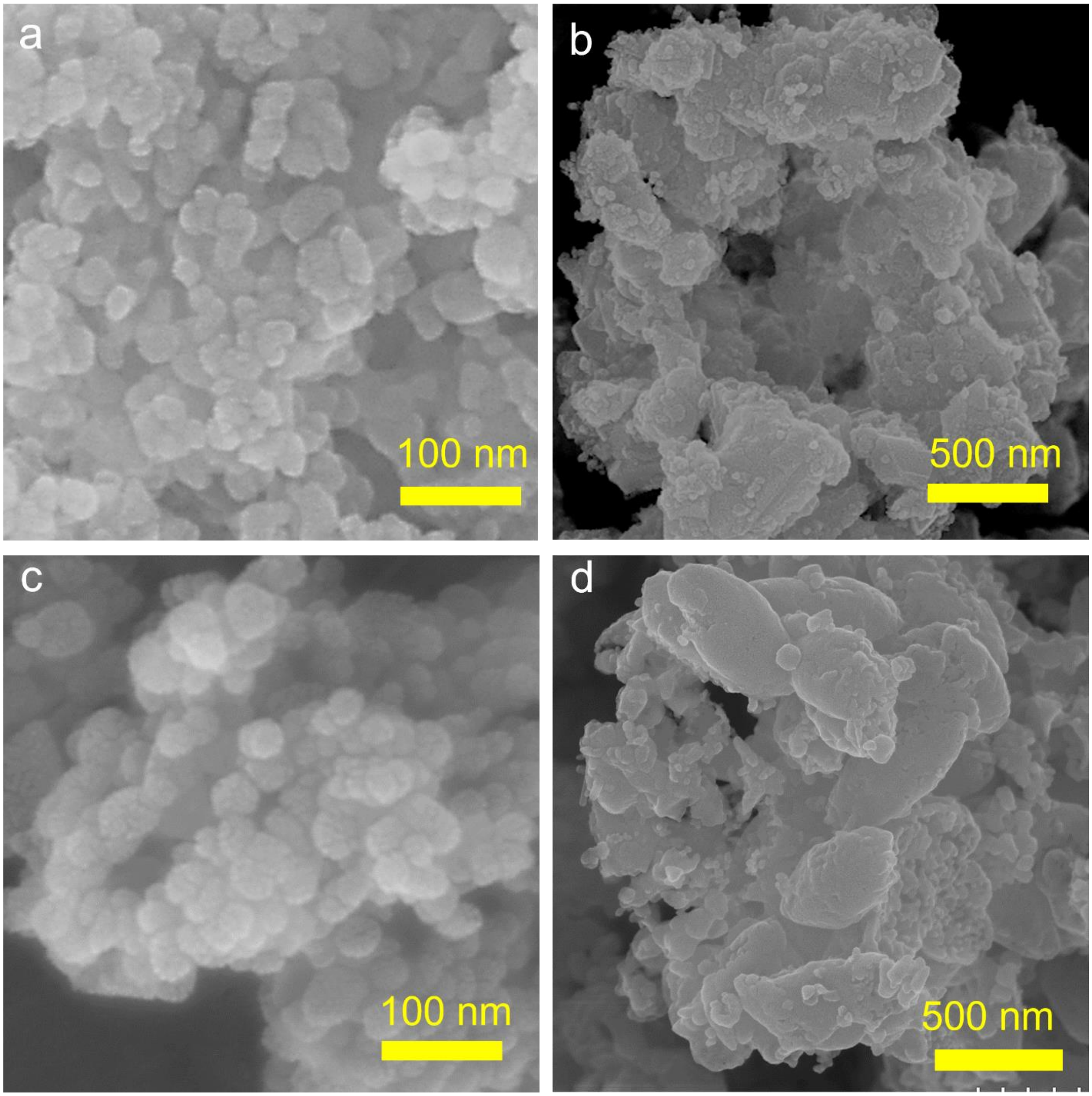

Figure S2 SEM images of different ESU materials: (a) fresh Fe/ZrO_2; (b) Fe/ZrO_2 after 100h testing; (c) fresh Fe/BZC4YYb; (d) Fe/BZC4YYb after 100 hours testing with U_{Fe}=50% and 0.2C at 550 ^oC.

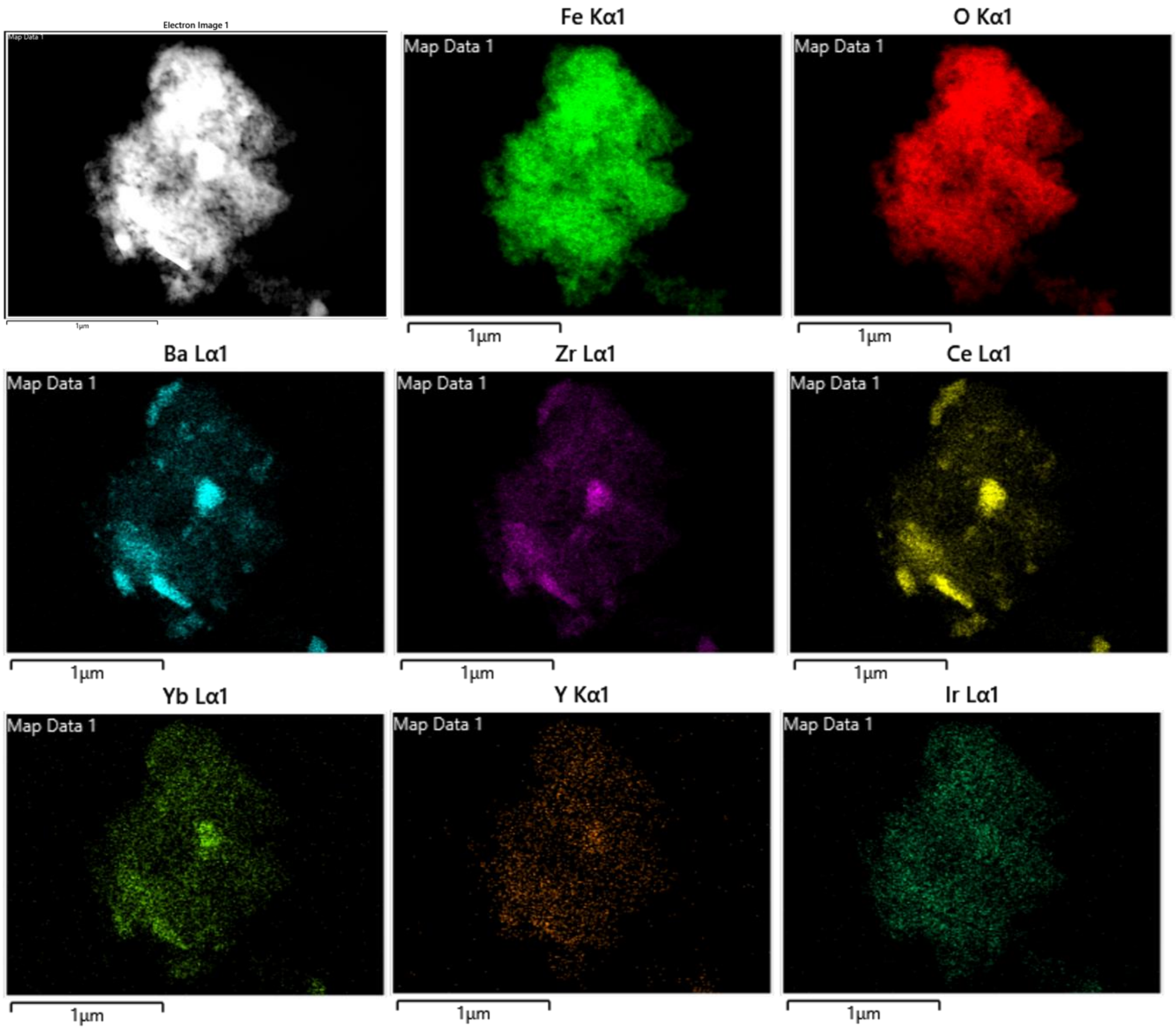

Figure S3 Element mapping for Fe, O, Zr, Ba, Ce, Y, Yb, Ir in Fe/BZC4YYb/Ir after 250 hours testing with U_{Fe}=50% and 0.2C at 550°C.

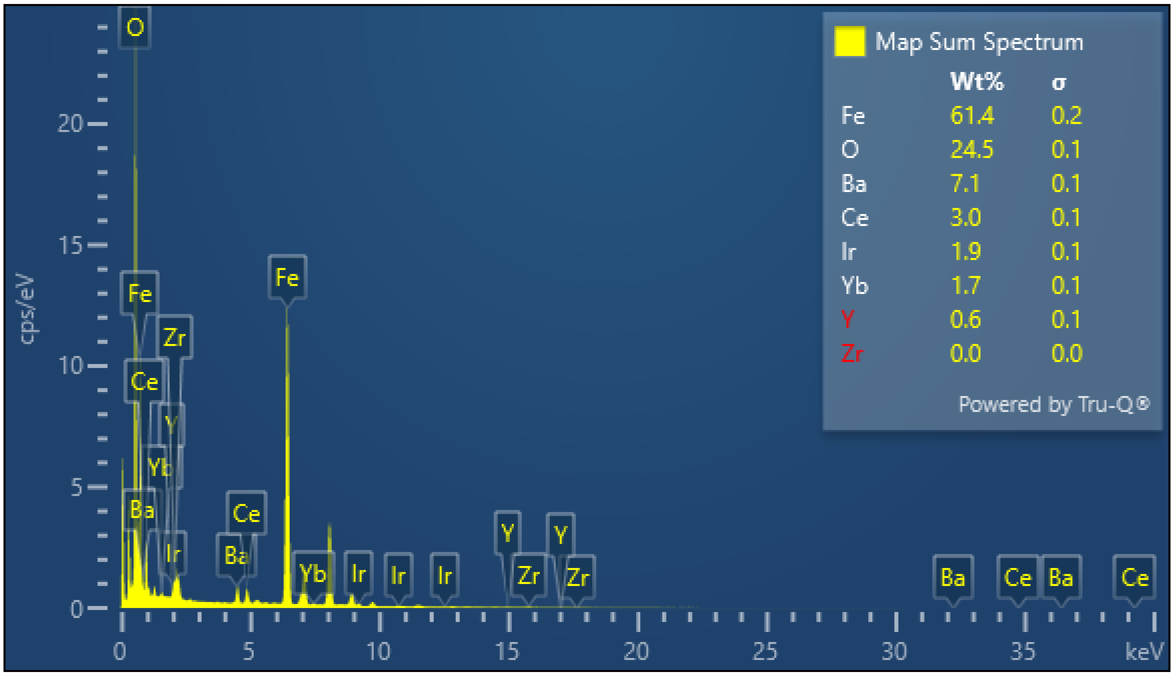

Figure S4 EDS Spectrum of Fe/BZC4YYb/Ir after 250-hour testing with U_{Fe}=50% and 0.2C at 550°C.

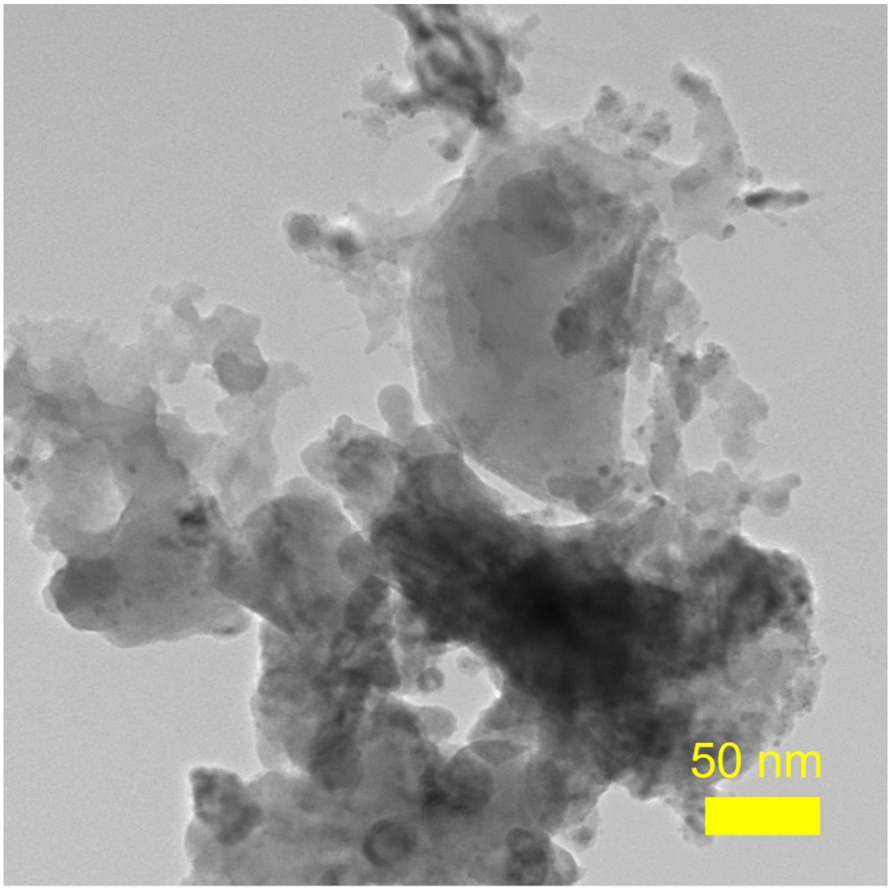

Figure S5 TEM images of Fe/BZC4YYb/Ir after 250 hours testing with U_{Fe}=50% and 0.2C at 550 ^{o}C .

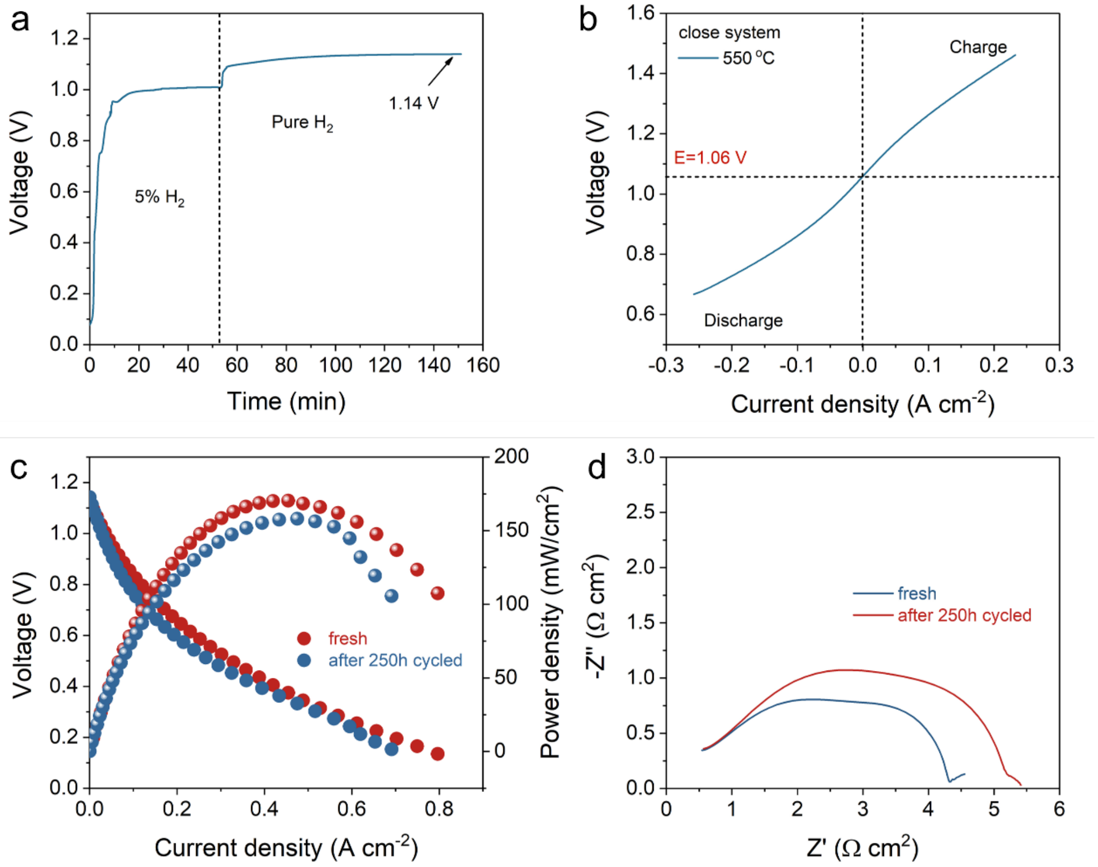

Figure S6 Electrochemical performance of RSOC in the battery with Fe/BZC4YYb/Ir ESU materials at 550°C: (a) profile of OCV *vs.* time during reduction process in open system; (b) V-I curve of battery in close system; (c) V-I and P-I curves and (d) EIS spectra measured in open system with H_2-3% H_2O as fuel before and after cycling testing.

Figure S6a shows the variation of OCV vs. time in H_2-3%H_2O in open system. An OCV of 1.14 V, near the theoretical potential, was achieved after fully reduction, implying a good gas tightness of the system. Then the performance of RSOC was also evaluated in a closed system, where the H_2 inlet and outlet were closed. Figure S6b shows the V-*j* curve in both fuel cell and electrolyzer modes in close system, where the OCV of battery is seen to decrease to ~1.06 V after closing the H_2 valve, signifying the equilibrium state of Fe and Fe_3O_4. The voltage is approximately linear with current density and no obvious concentration polarization even at a current density of j = 250 mA cm^{-2} at 550 °C, suggesting good electrochemical performance for

RSOC.

The power density and EIS of this HE-supported RSOC was examined in open system at 550 ^{o}C. The results shown in Figure S6c indicate a peak power density of 175 mW cm^{-2}, even after 250h cycling test at U_{Fe}=5% and 0.2C. A slight degradation of performance (~7.5%) is observed after the cycling. The EIS spectra shown in Figure S6d suggest an increase in low-frequency impedance, implying a possible HE-microstructure change. This is indeed confirmed by the increased concentration polarization observed at 0.6 A cm^{-2}.

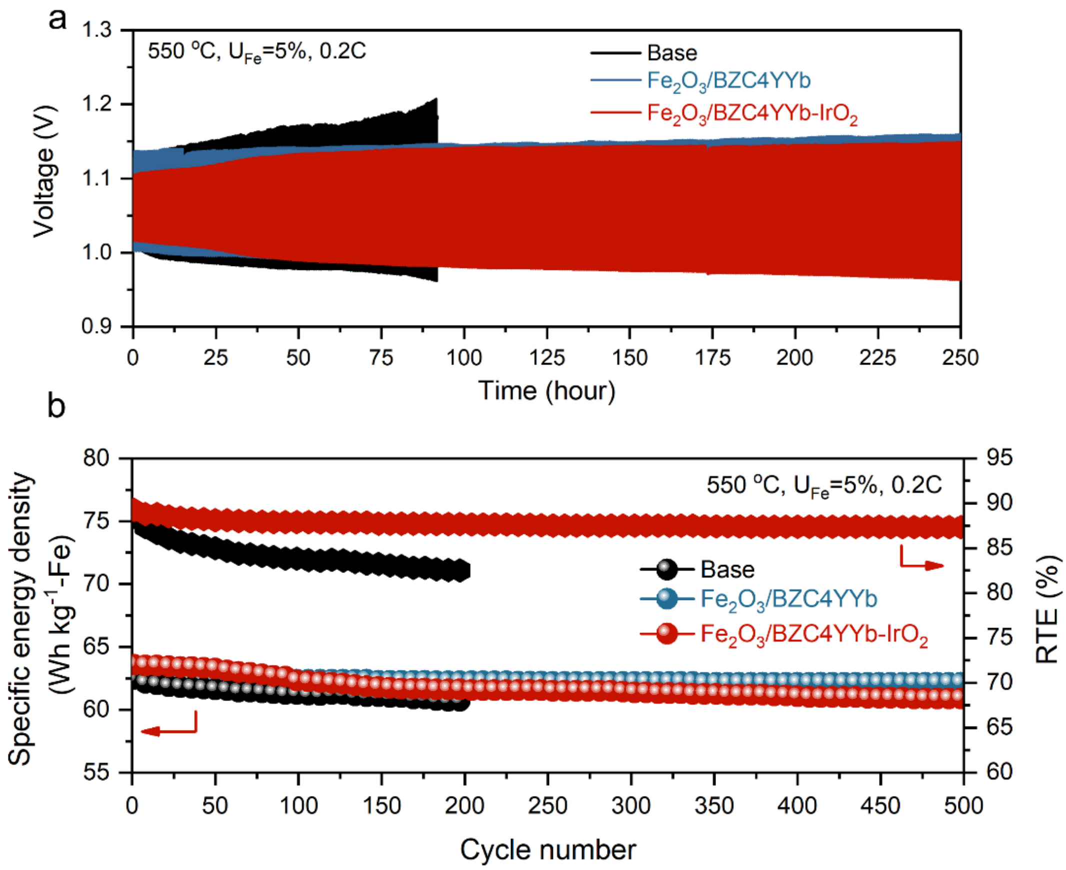

Figure S7. Cycling performance of a 550 °C battery with different ESU materials at U_{Fe}=5% and 0.2 C (10 mA cm^{-2}): (a) voltage profiles; (b) the corresponding SED and RTE.

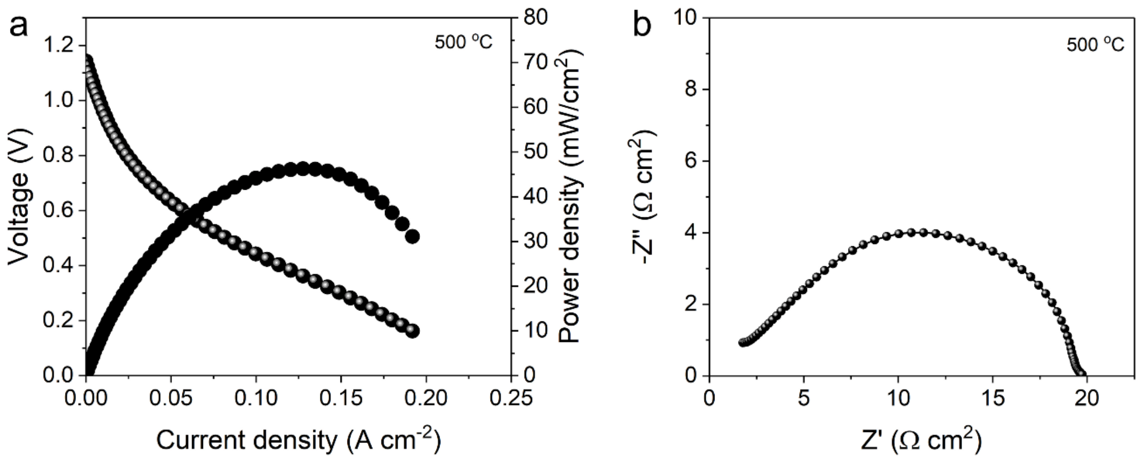

Figure S8 Electrochemical performance of RSOC of battery with Fe/BZC4YYb/Ir ESU materials at 500°C: (a) V-I and P-I curves and (b) EIS spectra measured under open circuit voltage in open system with H_2-3% H_2O as fuel before and after cycling testing.

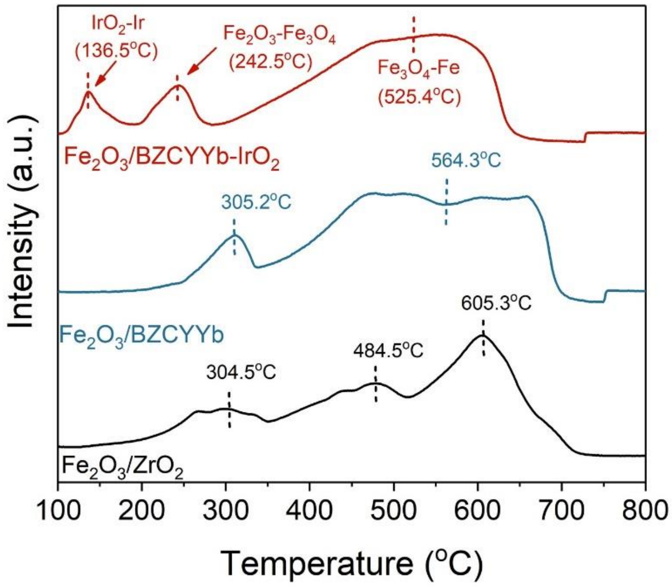

Figure S9. TPR profiles of different samples at a ramping rate of 10 °C/min: Fe/ZrO_2 (bottom); Fe/BZC4YYb (medium) and Fe/BZC4YYb/Ir (top); (b) Arrhenius plots of $\frac{\Phi}{{T_{max}}^2}$ for different materials.

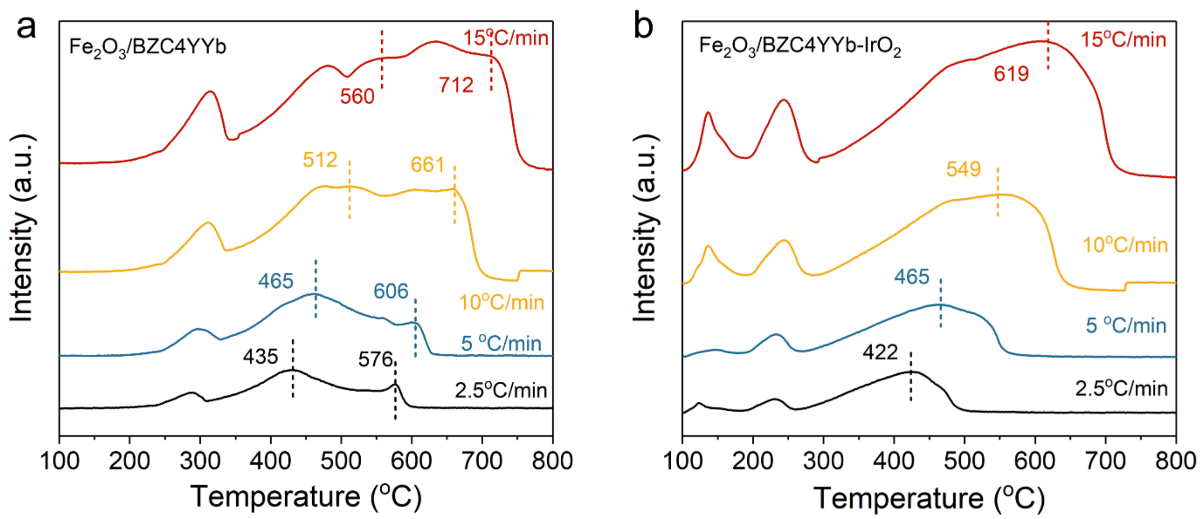

Figure S10 TPR profiles under different ramping rates for (a) Fe/BZC4YYb; (b) Fe/BZC4YYb/Ir.

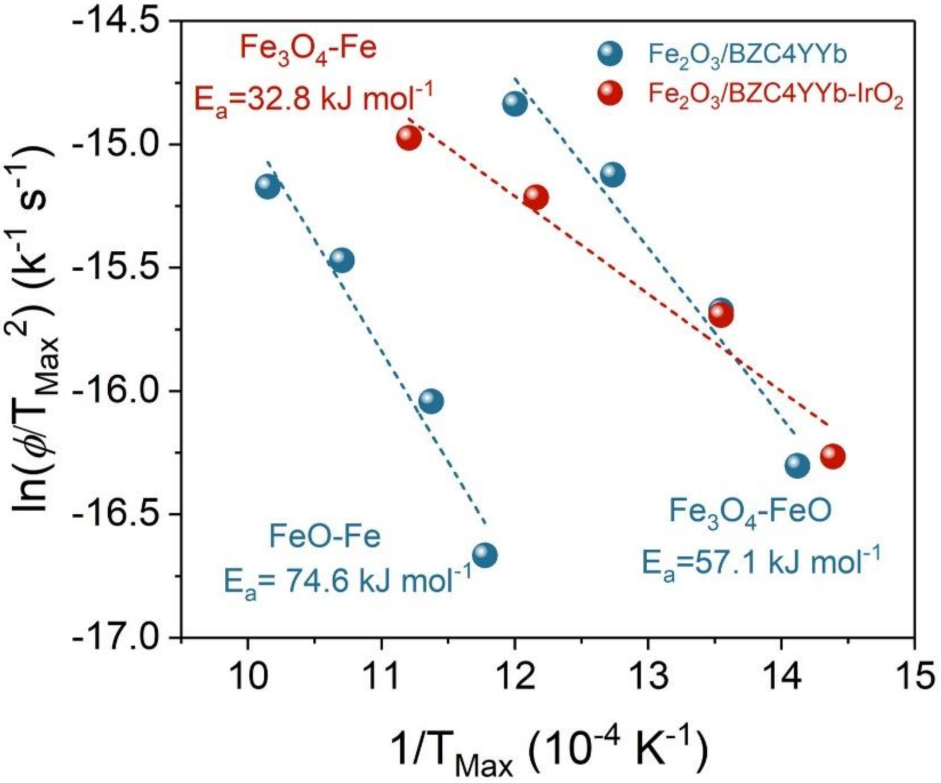

Figure S11. Arrhenius plots of $\frac{\Phi}{{T_{max}}^2}$ for Fe_2O_3/BZC4YYb and Fe_2O_3/BZC4YYb/Ir.
